# The uronic acid content of coccolith-associated polysaccharides provides insight into coccolithogenesis and past climate

**DOI:** 10.1038/ncomms13144

**Published:** 2016-10-26

**Authors:** Renee B. Y. Lee, Despoina A. I. Mavridou, Grigorios Papadakos, Harry L. O. McClelland, Rosalind E. M. Rickaby

**Affiliations:** 1 Department of Earth Sciences, University of Oxford, Oxford OX1 3AN, UK; 2 School of Biological Sciences, University of Reading, Whiteknights, Reading RG6 6UB, UK; 3 MRC Centre for Molecular Bacteriology and Infection, Department of Life Sciences, Imperial College London, London SW7 2DD, UK; 4 Department of Biochemistry, University of Oxford, Oxford OX1 3QU, UK

## Abstract

Unicellular phytoplanktonic algae (coccolithophores) are among the most prolific producers of calcium carbonate on the planet, with a production of ∼10^26^ coccoliths per year. During their lith formation, coccolithophores mainly employ coccolith-associated polysaccharides (CAPs) for the regulation of crystal nucleation and growth. These macromolecules interact with the intracellular calcifying compartment (coccolith vesicle) through the charged carboxyl groups of their uronic acid residues. Here we report the isolation of CAPs from modern day coccolithophores and their prehistoric predecessors and we demonstrate that their uronic acid content (UAC) offers a species-specific signature. We also show that there is a correlation between the UAC of CAPs and the internal saturation state of the coccolith vesicle that, for most geologically abundant species, is inextricably linked to carbon availability. These findings suggest that the UAC of CAPs reports on the adaptation of coccolithogenesis to environmental changes and can be used for the estimation of past CO_2_ concentrations.

## Abstract (web summary)

Coccolith-associated polysaccharides (CAPs) are central for the biomineralization of marine algae. Here, Lee *et al*. show that CAPs can be isolated from extant and fossil samples and that their uronic acid content can be used as a palaeoclimate proxy.

Calcifying coccolithophores form their intricate calcite structures within Golgi-derived intracellular vesicles and under the strict control of several components, such as an insoluble organic baseplate and water-soluble acidic polysaccharides^1^,^2^,^3^. The latter are known as coccolith-associated polysaccharides (CAPs) and they act as an organic framework for coccolith formation; they provide binding sites for the mineral components of calcification, nucleate specific crystallographic faces and dictate the rate of calcite precipitation *in vivo* and *in vitro*^4^,^5^,^6^,^7^. The ability to execute their role lies within their chemical composition^4^. CAPs are mainly composed of neutral monosaccharides complemented by two acidic moieties, sulphate esters and uronic acids^8^. It has been shown that it is uronic acids that provide the essential, negatively charged carboxyl groups that preferentially bind the calcium cations (Ca^2+^) (ref. 4) and initiate calcification.

CAPs are structurally and functionally diverse across different species of coccolithophores. Three CAPs are involved in the complex regulation of coccolithogenesis in *Pleurochrysis carterae*^9^ as opposed to one in *Emiliania huxleyi*^6^. Their biochemical composition has also been shown to vary across species^10^ and morphologically distinct strains of *E. huxleyi*^6^,^11^. In fact, this property was successfully exploited for antisera production that have been used for classifying *E. huxleyi* morphotypes A and B^12^.

CAPs do not single-handedly regulate coccolith biomineralization. Instead, they work in concert with the internal environment of the coccolith vesicle. Giuffre *et al*.^13^ demonstrated that the combination of polysaccharide chemistry with compartmental saturation state determines the kinetics of calcite nucleation. This means that CAP diversity is likely to be the adaptive outcome to a species-specific internal saturation state. This saturation state, given an abundance in calcium, and in species with a weak or absent carbon concentrating mechanism, is linked to the availability of carbon in the extracellular environment.

In this multidisciplinary study, we aim to elucidate the role of CAPs in dictating the morphological variation of coccoliths in relation to their adaptive strategy to changing CO_2_ concentrations over the millennia. For this reason, we sampled across a wide range of cultured strains and species and isolated CAPs from several sediment samples containing fossil coccoliths. In this way, we were able to shed light on the adaptation of CAPs through time and to propose their potential use as a species/strain biomarker and, more importantly, as a potential tool for inferring past environmental change.

## Results

### Coccolithophore samples from past and present

**Figure 1:**
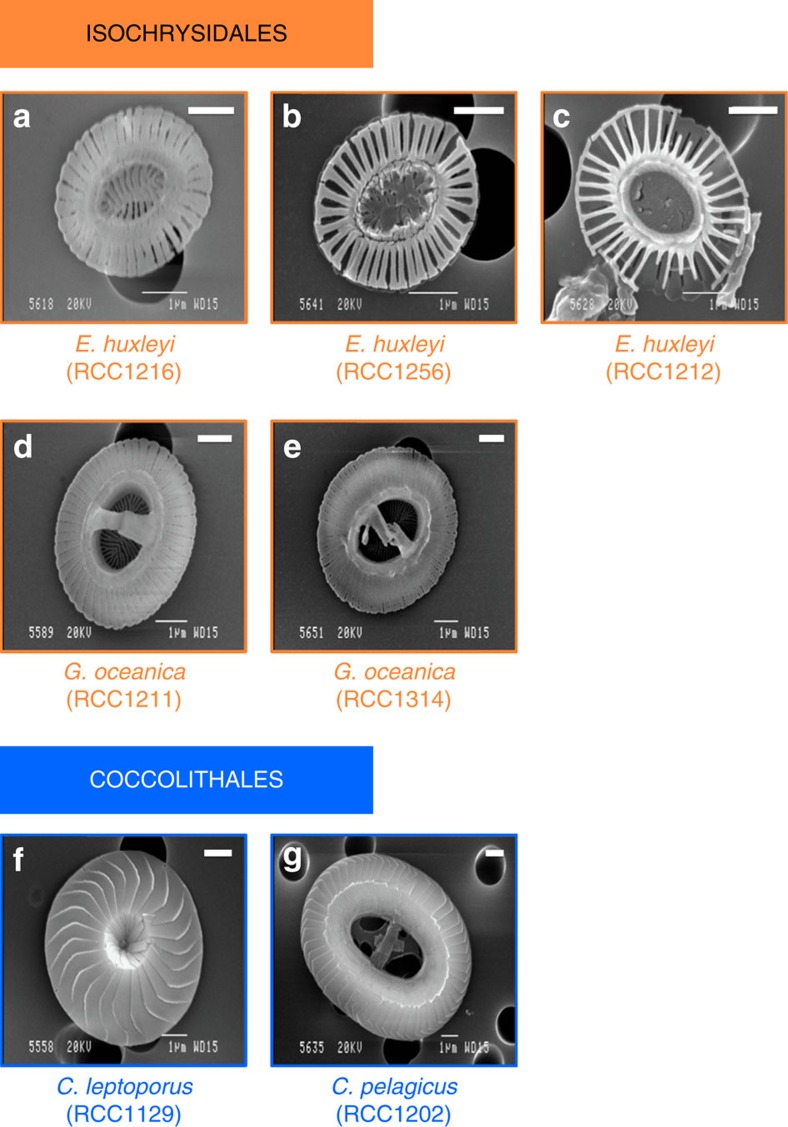
Scanning electron micrographs of coccoliths from live species. (**a**) *Emiliania huxleyi* (RCC1216, morphotype R); (**b**) *Emiliania huxleyi* (RCC1256, morphotype A); (**c**) *Emiliania huxleyi* (RCC1212, morphotype B); (**d**) *Gephyrocapsa oceanica* (RCC1211) (this strain was supplied from the culture collection as *Emiliania huxeyi*, morphotype B/C and was identified as *G. oceanica* in this study); (**e**) *Gephyrocapsa oceanica* (RCC1314); (**f**) *Calcidiscus leptoporus* (RCC1129); (**g**) *Coccolithus pelagicus* ssp. *braarudii* (RCC1202). Scale bars (top right), 1 μm.

In order to probe the diversity of CAPs, we cultured four extant species of coccolithophores. These are ultrastructurally and physiologically diverse organisms, present in a variety of habitats and either have identifiable ancestors or are themselves abundant in the geological record. *Coccolithus pelagicus* ssp. *braarudii* (family Coccolithaceae) (Fig. 1g) and *Calcidiscus leptoporus* (family Calcidiscaceae) (Fig. 1f) are two of the largest and most heavily calcifying species from the order Coccolithales. This order diverged from its last common ancestor with the Isochrysidales after the P/Tr boundary (∼243 Ma; ref. 14), and has prevailed throughout the early Cenozoic, only to be marginalized in present-day oceans^15^. *Gephyrocapsa oceanica* (Fig. 1d,e) and *E. huxleyi* (family Noëlaerhabdaceae) (Fig. 1a–c), were selected from the geologically younger order Isochrysidales. The genus *Gephyrocapsa* first appeared in the late Pliocene (∼3.5 Ma) and rose to dominance in the Early Pleistocene^16^. Its descendant species, *E. huxleyi* (at ∼250 ka), has since become the most abundant coccolithophore in the modern ocean^1^. Strains of *E. huxleyi* representing three distinct morphotypes with varying degrees of calcification as well as subtle ultrastructural differences, were selected for this study (Fig. 1a–c, Supplementary Table 1). The degree of coccolith calcification of the *E. huxleyi* morphotypes can be arranged in the order of B (Fig. 1c)<A (Fig. 1b)<R (Fig. 1a) based on morphometric observations^17^,^18^,^19^.

Recently, Sand *et al*.^20^ demonstrated that CAPs are preserved in chalk deposits from the Maastrichtian (65–70 Ma). These ancient extracts were still active and displayed the characteristic properties of known CAPs^5^,^20^. For this reason we wanted to analyse the properties of CAPs not only from cultured samples but also from fossil coccoliths. We obtained sedimentary material from the Upper Pleistocene (∼107 ka), as well as the geologically older Serravalian (12.4–13.6 Ma), Langhian (∼14.5 Ma), Burdigalian (∼16 Ma), Campanian (76–80 Ma), Late Cenomanian (∼94 Ma), Tithonian (∼150 Ma) and Early Toarcian (∼180 Ma) (Supplementary Table 2). The fine-fraction from the Upper Pleistocene was separated into mono-specific coccolith fractions; the three size-delimited fractions retained for analysis consist of the *C. pelagicus* fraction (10–12 μm), the Noëlaerhabdaceae fraction (0–3 μm) and the bulk coccolith fraction (<12 μm), which contains all contemporaneous coccoliths from this site (cross-polarized micrographs of these fractions are shown in Supplementary Fig. 1a). By mass of calcite, over 90% of the coccoliths in the 10–12 μm fraction were *C. pelagicus* and ∼30% of the total calcite across all size fractions was foraminiferal debris. The quality of separation in terms of relative numerical abundance by species is summarized in Supplementary Table 3. Samples from the Serravalian, Langhian and Burdigalian are almost mono-specific coccolith fractions with coccolith sizes larger than 8 μm, which predominantly consist of *Discoaster* spp. and *C. pelagicus* (a representative cross-polarized micrograph is shown in Supplementary Fig. 1b). Samples from the Campanian, Late Cenomanian, Tithonian and Early Toarcian were analysed in bulk. Fossils from the Late Cenomanian and Early Toarcian mainly consist of the extinct *Watznaueria* spp. and *Crepidolithus* spp., respectively while samples from the Campanian and Tithonian also predominantly consist of larger coccolithophores (predecessors of the modern Coccolithales).

### Isolation and biochemical characterization of CAPs

**Figure 2:**
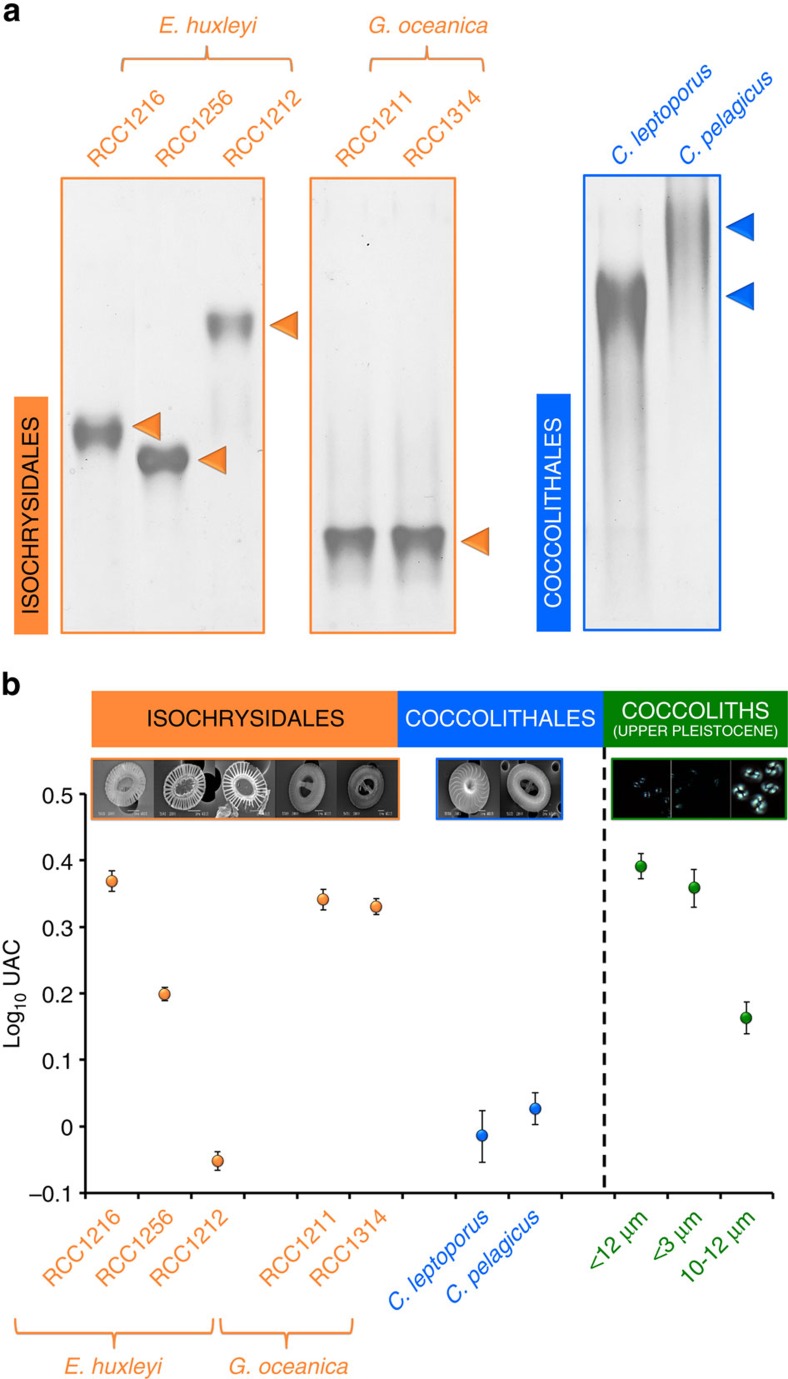
The size and uronic acid content of coccolith-associated acidic polysaccharides provide a species-specific signature. (**a**) Polyacrylamide gel electrophoresis (PAGE) of CAPs isolated from cultured coccolithophores. Analysis was carried out using 4 μg of each coccolith-associated acidic polysaccharide (CAP) on a 12% polyacrylamide gel. CAPs were stained with Alcian Blue. One CAP was isolated from each species/strain; this is obvious from the presence of a single band in each lane (indicated by an orange or blue arrowhead). (**b**) Variation in the uronic acid content (UAC) of CAPs extracted from cultured coccolithophores and fossil coccoliths. The UAC of CAPs from present day species/strains and size-separated fractions from the ODP1123 geological site (Upper Pleistocene, South Pacific Ocean) is plotted on a logarithmic scale. Error bars represent the s.d. between two independent replicates. The exact average UAC values for each sample are given in Supplementary Table 6.

We successfully isolated the CAPs from several cultured samples and also from sediment samples dating back to ∼180 Ma (Supplementary Table 4) and initially tested the extracts for the presence of protein. We subjected the CAPs to native polyacrylamide gel electrophoresis (PAGE) and stained the gel with Coomassie Brilliant Blue R-250 that would stain any protein molecules, however, no bands were visible. Similarly, no protein was detected by Bradford assay. Although these tests suggest the absence of protein molecules in our extracts, we cannot preclude the role of proteins in coccolith biomineralization^6^,^21^. Subsequently, we stained the gel with Alcian blue, a dye that selectively complexes the carboxyl groups of acidic polysaccharides (Fig. 2a). A single band was detected for each extract (indicated by an orange or blue arrowhead in Fig. 2a) and this demonstrated the isolation of one CAP per strain/species. Although all CAPs migrated as a single band, we observed a significant variation in their electrophoretic mobility (Fig. 2a). We subjected the same samples to denaturing sodium dodecyl sulfate (SDS)–PAGE analysis (Supplementary Fig. 2). The presence of the anionic detergent SDS imparts a uniform negative charge on the CAPs during SDS–PAGE analysis so that the polysaccharides migrate only according to their size. Since the migration pattern of the CAPs is identical during PAGE and SDS–PAGE analysis (Supplementary Fig. 2) we can conclude that the observed migration pattern of CAPs during PAGE-analysis is not affected by their charged (sulfonic and uronic acid) residues. Therefore, there is a true difference in the size of our isolated CAPs, which concurs with previous reports^6^,^10^.

CAPs from cultured and fossil samples were also subjected to reverse-phase high-performance liquid chromatography (RP-HPLC) to confirm their identity as acidic polysaccharides (Supplementary Fig. 3a). Apple pectin (polygalacturonic acid), a known acidic polysaccharide that has been shown to promote calcification^13^, was also subjected to RP-HPLC analysis as a positive control (Supplementary Fig. 3b). We found that all tested CAPs have the same polarity as apple pectin because they eluted in the same solvent gradient range (marked by the grey shaded area in Supplementary Fig. 3a,b). This confirmed that our extracts are indeed acidic polysaccharides. Interestingly, we also observed that our CAPs, as well as pectin, elute as multiple peaks during RP-HPLC analysis. This demonstrates the occurrence of several potential spatial configurations (conformers) of each CAP molecule. Size-exclusion chromatography showed the same; a single but broad peak was observed for each extract confirming the presence of more than one conformer for each extracted CAP (see the representative example of the analysis of the *G. oceanica* RCC1314 CAP in Supplementary Fig. 3c; the peak of interest is marked by the grey shaded area). It is important to note that identical amounts of CAPs from cultured and fossil samples gave nearly identical traces, in shape, intensity and elution time, during RP-HPLC analysis (Supplementary Fig. 3a). Had degradation of the CAPs that we extracted from fossils coccoliths taken place, we would have recorded very different elution profiles for these extracts compared with the CAPs that we isolated from live organisms. Therefore, we can conclude that the CAPs we isolated from fossil samples are intact and very similar in chemical composition to the CAPs from modern cultures containing the same species/strains. Preservation of CAPs in fossils is also in agreement with previous reports of CAP extraction from sediments^20^,^22^. We note that PAGE (or SDS–PAGE) and RP-HPLC analysis, although overall very informative, are mostly qualitative characterization methods. For a more accurate measure of the relative size differences between different CAPs and of the exact degree of preservation of CAPs extracted from fossil samples, size-exclusion chromatography, followed by reduction of the samples and mass spectrometry would probably be advantageous.

### UAC of CAPs as a species-specific biomarker

By providing the negatively charged carboxyl groups that interact with the positively charged Ca^2+^ in the coccolith vesicle, uronic acid moieties in CAPs are crucial in dictating the overall calcite nucleation and precipitation rates^6^. For this reason, we measured the uronic acid content (UAC) for all our extracts from cultured and fossil samples using a modified carbazole-H_2_SO_4_ assay^23^ (Fig. 2b, Supplementary Table 6). Our results show that the UAC varies significantly between species/strains and this fluctuation can be successfully used for biochemical distinction between strains of *E. huxleyi* and across species of coccolithophores (see the example mentioned in Supplementary Table 1). The UAC-species relationship is conserved in CAPs isolated from sediments, with the Coccolithales (10–12 μm fraction) consistently yielding lower UAC than the Isochrysidales (<3 μm and dominating the mixed <12 μm). Thus, based on the data presented in Fig. 2b, the UAC of CAPs can be used to complement existing tools for diagnosing taxonomic affinities when morphological and DNA preservation is poor or ambiguous^24^,^25^. Furthermore, the UAC is a sensitive enough biomarker, able to distinguish even between different clonal strains of *E. huxleyi*^11^. This metric could be used for determining clonal distribution and detecting population shifts in the environment, which is key to the understanding of the adaptive potential of *E. huxleyi* populations and their impact on biogeochemical cycles.

### UAC of CAPs and saturation state of the coccolith vesicle

According to Fig. 2b, the UAC of CAPs that were isolated from Isochrysidales is directly proportional to their recorded degree of calcification as depicted in our scanning electron microscopy (SEM) images (Fig. 1a–e). In strains of *E. huxleyi*, the CAP of RCC1216 (morphotype R, Fig. 1a), which is frequently described as heavily calcifying, has the highest UAC, whereas the lightly calcified coccoliths of RCC1212 (morphotype B, Fig. 1c) have the lowest. The same correlation can be made for *G. oceanica*; both strains we tested have a high UAC (Fig. 2b) and are heavily calcifying (Fig. 1d,e). However, an inverse relationship is observed in Coccolithales, where low UAC did not result in a lightly calcified coccolith (Figs 1f,g and 2b). This observation seems paradoxical at first but it can be explained if one takes into account the charge density of CAPs in relation to the saturation state of the coccolith vesicle and its effect on the nucleation rate of calcite. It is important to remember that, according to Giuffre *et al*.^13^, substrates rich in carboxyl functional groups (high UAC in the case of CAPs) promote fast nucleation rates at high intracellular supersaturation, whereas at low saturation states, substrates with lower charge density (low UAC) promote faster rates of nucleation. Unfortunately, due to the transient nature of the coccolith vesicle, there is little information pertaining to its chemical state during coccolithogenesis^26^. More specifically, in order to generate an absolute measure of internal saturation state we would need to know the pH and the concentration of Ca^2+^ in the coccolith vesicle during calcification; both these parameters are currently unknown. However, by considering the published data on several contributing factors known to affect the internal saturation state (for example, the utilization rates of calcium and carbon, the supply of carbon and the size of the organism), we can predict if a coccolithophore is likely to have a high or low saturation state in its coccolith vesicle.

In Supplementary Table 5 we present all the published information on parameters that are known to affect the internal saturation state of coccolithophores. The high utilization rate of calcium over carbon (given by the high PIC/POC, where PIC stands for particulate inorganic carbon and POC for particulate organic carbon), the slow CO_2_ diffusion (given by the low SA/*V*, where SA is the surface area and *V* is the volume of the coccolithophore) and the large size of the Coccolithales like *C. pelagicus*, point towards a low internal saturation state of this order. This is further supported by the lack of any evidence that the Coccolithales have a carbon concentrating mechanism and the fact that they are generally characterized by a low internal dissolved inorganic carbon pool^27^,^28^. Therefore, their measured low UAC (Fig. 2b) correlates very well with efficient nucleation at lower internal saturation state that results in a highly calcified coccolith (Fig. 1f,g). On the other hand, Isochrysidales have a lower utilization rate of calcium over carbon (lower PIC/POC), they are significantly smaller (approximately fivefold smaller in diameter) (Supplementary Table 5) and they have a high SA/*V* ratio, which means that they acquire CO_2_ from their environment much more efficiently than the Coccolithales. In addition, there is evidence for a carbon concentrating mechanism in the ability of members of this order to utilize their dissolved inorganic carbon to increase their internal saturation state^29^,^30^,^31^,^32^,^33^. All the above contribute towards a high saturation state in the coccolith vesicle of Isochrysidales, which means that optimal calcification is achieved by high charge densities (high UAC for the heavily calcifying species/strains as shown in Fig. 2b). We can, therefore, conclude that by altering their polysaccharide charge density, coccolithophores are able to fine-tune their calcite precipitation process to varying degrees of internal supersaturation and that this adaptation, in most cases, allows them to achieve optimal calcification.

### UAC of CAPs and environmental *p*CO_2_

**Figure 3:**
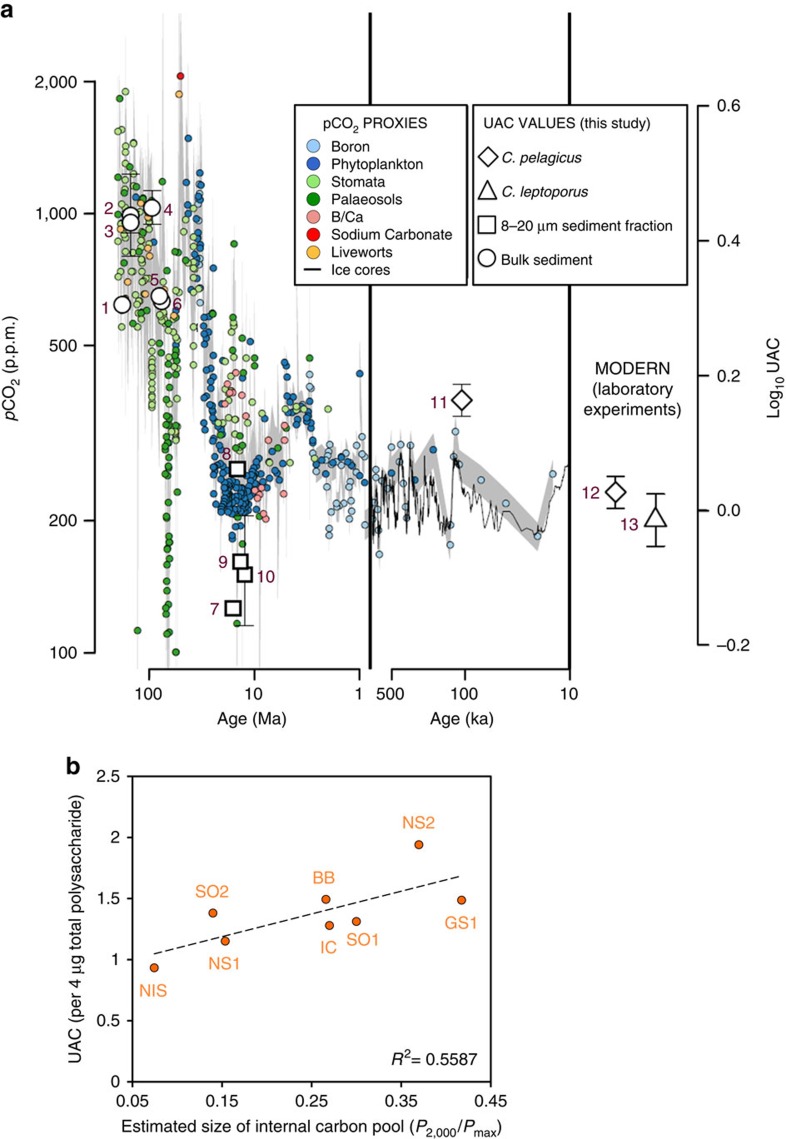
The uronic acid content of coccolith-associated acidic polysaccharides correlates with the size of the internal carbon pool of the coccolithophore. (**a**) Average uronic acid content (UAC) values of coccolith-associated acidic polysaccharides (CAPs) isolated from modern Coccolithales (points 12 and 13), *C. pelagicus* from the Upper Pleistocene sediments (point 11) or the Neogene sediments (points 7–10) and prehistoric predecessors of the Coccolithales from bulk fossil samples (points 1–6). The UAC values are plotted alongside proxy-reconstructed atmospheric *p*CO_2_ for the past 200 Ma and ice core CO_2_ records. The grey lines represent the range of *p*CO_2_ as estimated from proxies and ice core data (at 1σ confidence level). Proxies were compiled from Royer^44^, Park and Royer^45^, Beerling and Royer^46^ and Hönisch *et al*.^47^; ice core CO_2_ records were obtained from Lüthi *et al*.^48^. Details on the cultured and fossil samples can be found in Supplementary Tables 1 and 2, respectively and the exact UAC values for each point are given in Supplementary Table 7. Error bars represent the s.d. between two independent replicates. (**b**) UAC (per 4 μg total CAP) of the CAPs extracted from recently isolated strains of *Emiliania huxleyi* (morphotype A and B/C) versus the estimated size of the intracellular carbon pool (inferred from the ratio of photosynthetic rates at near ambient conditions of 2,000 μmol kg^−1^ dissolved inorganic carbon (*P*_2,000_) and the maximum rate of photosynthesis (*P*_max_)) (as detailed in Rickaby *et al*.^11^). Details on the *E. huxleyi* strains (abbreviated in orange on the graph) and the exact UAC, *P*_2,000_ and *P*_max_ values for each point are given in Supplementary Table 8.

Characterization of CAPs extracted from the geological record lends further support to this proposal. Isochrysidales and Coccolithales have distinct geological histories with regard to cell size^15^,^34^,^35^. Estimates based on coccolith morphometrics suggest that the cell size of *Coccolithus* spp. and *Calcidiscus* spp. remain largely unchanged throughout the Cenozoic decline in *p*CO_2_ (ref. 36). We found that CAPs extracted from older Coccolithale-containing fossil samples from the greenhouse high *p*CO_2_ world have a high UAC (Fig. 3a, Supplementary Table 7; points 1–6), which was beneficial for calcification when carbon availability and the internal carbon pool of the cell was high. However, in order to sustain calcification as their internal pool diminished during the decline in *p*CO_2_ and in the absence of evolution of a strong carbon concentrating mechanism, more recent Coccolithales reduced their CAP charge in favour of retaining a larger cell size (Fig. 3a, Supplementary Table 7; points 7–13). Isochrysidales, on the other hand, have been shown to diminish their size through time as a form of adaptation to declining *p*CO_2_ (ref. 37). As such, we found that they have overall a relatively high UAC that promotes calcification in a consistently highly saturated environment (Fig. 2b, Supplementary Table 6).

The UAC values of CAPs we extracted from recently isolated *E. huxleyi* strains from different locations provide additional evidence for the relationship between UAC and internal carbon pool^11^. We measured the photosynthetic parameters *P*_2,000_ and *P*_max_ of eight recently isolated strains using an oxygen electrode; *P*_2,000_ is the photosynthetic rate at ambient CO_2_ availability and *P*_max_ is the maximum photosynthetic rate, which correlates with the CO_2_ availability at the site of isolation of each strain. The ratio of these two parameters provides a measure of the internal carbon pool (as explained in detail in Rickaby *et al*.^11^). We observed that high UAC content correlates with a large internal carbon pool (that is, high *P*_2,000_/*P*_max_) (Fig. 3b, Supplementary Table 8 adapted from Rickaby *et al*.^11^). Therefore, it seems that *E. huxleyi*, have stopped varying in size (having reached a potential size minimum) in the modern ocean^35^, and instead they fine-tune their UAC content according to *p*CO_2_ availability in their local environment in order to achieve optimal calcification. This modern day adaptation would be equivalent to the geological strategy adopted over the millennia by the Coccolithales that have not changed in size and instead have been modulating their UAC in order to calcify efficiently despite the drop in their internal saturation state.

In conclusion, we propose CAPs as a promising new tool for the field of biogeochemistry. They are ancient molecules that, until today, have the same clear biological involvement in the calcification process of coccolithophores as they had millions of years ago^5^,^20^,^22^. They are preserved through time and can be successfully extracted in pure form from fossils and live cultures (Fig. 2a, Supplementary Figs 2 and 3). In addition, the UAC of CAPs can be used as a palaeoclimate proxy because it correlates with the internal saturation state of the coccolith vesicle and, for species with a weak or absent carbon concentrating mechanism, essentially reports on the change in environmental *p*CO_2_ (Figs 2b and 3). Ultimately, this is almost a unique case where a biological signal goes hand-in-hand with climate change and provides us with an invaluable glimpse into the past.

## Methods

### Cultures and fossil material

Live species/strains used in this study were obtained from the Roscoff Culture Collection (www.sb-roscoff.fr/Phyto/RCC) and are listed in Supplementary Table 1. Cultures were grown in aged, filter-sterilized (0.22 μm Stericup-GP Filter Unit, Millipore) seawater enriched with trace metals, nitrate, phosphate and vitamins according to Probert and Houdan^38^, under 150 μmol m^−2^ s^−1^ illumination (12/12 h light/dark cycle) at 15 °C.

Fossil samples used in this study are listed in Supplementary Table 2. Fossil coccoliths from the last glacial inception (∼107 ka) were isolated from sediments from the ocean drilling project (ODP) Site 1,123 in the Southern Pacific Ocean. A 30 g-fraction representing a time slice of ∼1,000 years was separated into mono-specific coccolith fractions according to Minoletti *et al*.^39^. The <12 μm, 10–12 and 0–3 μm fractions from this site were retained for further analysis. The same separation method was used to generate near mono-specific fractions of fossil coccoliths from the sediments obtained from the deep sea drilling project (DSDP) Site 588 in the Southwest Pacific. Four fractions of different age were retained (8–20 μm (∼12.4 Ma); 12–20 μm (∼13.6 Ma); 8–10 μm (∼14.5 Ma); 10–12 μm (∼16.0 Ma)). Smear slides containing these samples were prepared and viewed under a cross-polarized light microscope and approximate relative numerical species abundances were determined by manual counts (Supplementary Table 3 and Supplementary Fig. 1). Bulk sediments from the Campanian, Late Cenomanian, Tithonian and Early Toarcian were pre-crushed and subsequently disaggregated in neutralized water by immersion in an ultrasonic bath (30 W for ∼1 h). The final suspension was allowed to settle and the supernatant was removed. Experiments described above were carried out in the Department of Earth Sciences (University of Oxford).

### Scanning electron microscopy

Samples for SEM were filtered onto polycarbonate filters (2.0 μm pore size), rinsed with de-ionized water (pH 7.0), dried at room temperature for 24 h, and sputter coated with gold–palladium. Imaging was performed with a Jeol JSM-840A SEM at 20 kV. Experiments described above were carried out in the Department of Earth Sciences (University of Oxford).

### Extraction of polysaccharides

CAPs were extracted from cultured and sediment samples by modification of several protocols^22^,^40^,^41^. Harvested cells and sediments were cleaned with 1% v/v Triton X-100 and 4.5% v/v NaOCl in 0.05 M NaHCO_3_. After the removal of organically bound matter, the samples were rinsed thoroughly with ultrapure water and stored at −80 °C. Before extraction, the coccolith pellet was resuspended in 0.05 M NH_4_HCO_3_ by ultrasonication and the resulting suspension was further purified by centrifugation through a gradient of 100 ml Ludox TM-50 colloidal silica layered with 25 ml 20% w/v sucrose, at 23,000*g* for 20 min at 4 °C. The supernatant was discarded and the pellet was washed five times with 0.05 M NH_4_HCO_3_. Subsequently, the pellet was decalcified by incubating for 12 h in 0.5 M EDTA (pH 8.0) at 4 °C, followed by ultrasonication. Insoluble residues were removed by centrifugation at 31,000*g* and the supernatant was buffer-exchanged into 20 mM Tris HCl (pH 8.0) using a 10,000 MWCO Amicon Ultra-4 membrane (Millipore). The sample was then subjected to anion exchange liquid chromatography using a HiTrap DEAE FF (GE Healthcare) according to the manufacturer's instructions. The coccolith acidic polysaccharide (CAP) was eluted using 20 mM Tris.HCl (pH 8.0), 0.5 M NaCl, buffer-exchanged into ultrapure water and stored at −20 °C. Experiments described above were carried out in the Department of Earth Sciences (University of Oxford).

### Quantification of total polysaccharide and uronic acid

Polysaccharides were quantified using a phenol-H_2_SO_4_ assay^42^ with glucose as a standard. The UAC was determined using a modified carbazole-H_2_SO_4_ assay^23^ with glucuronic acid as a standard. The values obtained were normalized to 4 μg total polysaccharide. Experiments described above were carried out in the Department of Earth Sciences (University of Oxford).

### Electrophoresis of polysaccharides

Isolated CAPs were subjected to 12% PAGE and SDS–PAGE. 4 μg of CAP were loaded on each lane. Alcian blue staining was carried out according to Møller *et al*.^43^. Briefly, gels were rinsed in wash solution 1 (25% v/v ethanol and 10% v/v acetic acid) for 1.5 h at 50 °C and, subsequently, stained with 0.1% w/v Alcian Blue 8 GX in wash solution 1 for 15 min at 50 °C. The gels were de-stained by firstly rinsing with wash solution 1 for 15 min at 50 °C, and then with wash solution 2 (10% v/v ethanol, 5% v/v acetic acid) for 1.5 h at 50 °C. Experiments described above were carried out in the Department of Earth Sciences (University of Oxford).

### Chromatographic analysis of polysaccharides

The samples (100 μg of extracted polysaccharide) were analysed by RP-HPLC using a Vydac C8 column (4.6 by 250 mm; 5 μm particle size) without a guard column on an Äkta purifier system (GE Healthcare). Samples were centrifuged at 16,000*g* for 5 min to remove any insoluble material before the chromatography step. The elution buffers were: (A), 0.1% trifluoroacetic acid in ultrapure water; (B), 0.1% trifluoroacetic acid in acetonitrile. The column was equilibrated in buffer (A) at 2 ml min^−1^ at room temperature before injection of the sample. The running method involved the following steps: (1) linear increase to 20% buffer (B) over 4 min that was initiated at the moment of injection; (2) isocratic elution at 20% buffer (B) for 5 min; (3) linear increase to 40% buffer (B) over 5 min; (4) isocratic elution at 40% buffer (B) for 5 min; (5) linear increase to 100% buffer (B) over 7.5 min. The eluted compounds were detected by monitoring the absorbance of the polysaccharides at 230 nm. The grey shaded areas of the eluted peaks (Supplementary Fig. 3) correspond to steps 3–5 of the above method. Fifty micrograms of apple pectin (76,282, Sigma-Aldrich) were analysed by RP-HPLC, as above. The size exclusion chromatographic analysis of the *G. oceanica* RCC1314 CAP, was carried out by applying 100 μg of polysaccharide to a Superdex 200 PC 3.2/30 column (GE Healthcare) pre-equilibrated in 50 mM NaCl at 0.1 ml min^−1^. Experiments described above were carried out in the Department of Department of Biochemistry (University of Oxford).

### Data availability

All data supporting the findings of this study will be made available by the authors upon request.

## Additional information

**How to cite this article:** Lee, R. B. Y. *et al*. The uronic acid content of coccolith-associated polysaccharides provides insight into coccolithogenesis and past climate. *Nat. Commun.* **7,** 13144 doi: 10.1038/ncomms13144 (2016).

**Publisher's note:** Springer Nature remains neutral with regard to jurisdictional claims in published maps and institutional affiliations.

## Supplementary Material

Supplementary Information Supplementary Figures 1-3, Supplementary Tables 1-8, and Supplementary References
