# Supplementary material for: The uronic acid content of coccolith-associated polysaccharides provides insight into coccolithogenesis and past climate
Source: Nat Commun. 2016 Oct 26;7:13144. doi: 10.1038/ncomms13144 (PMC5095175; doi:10.1038/ncomms13144)
Supplement: Supplementary Information — Supplementary Figures 1-3, Supplementary Tables 1-8, and Supplementary References [file ncomms13144-s1.pdf]

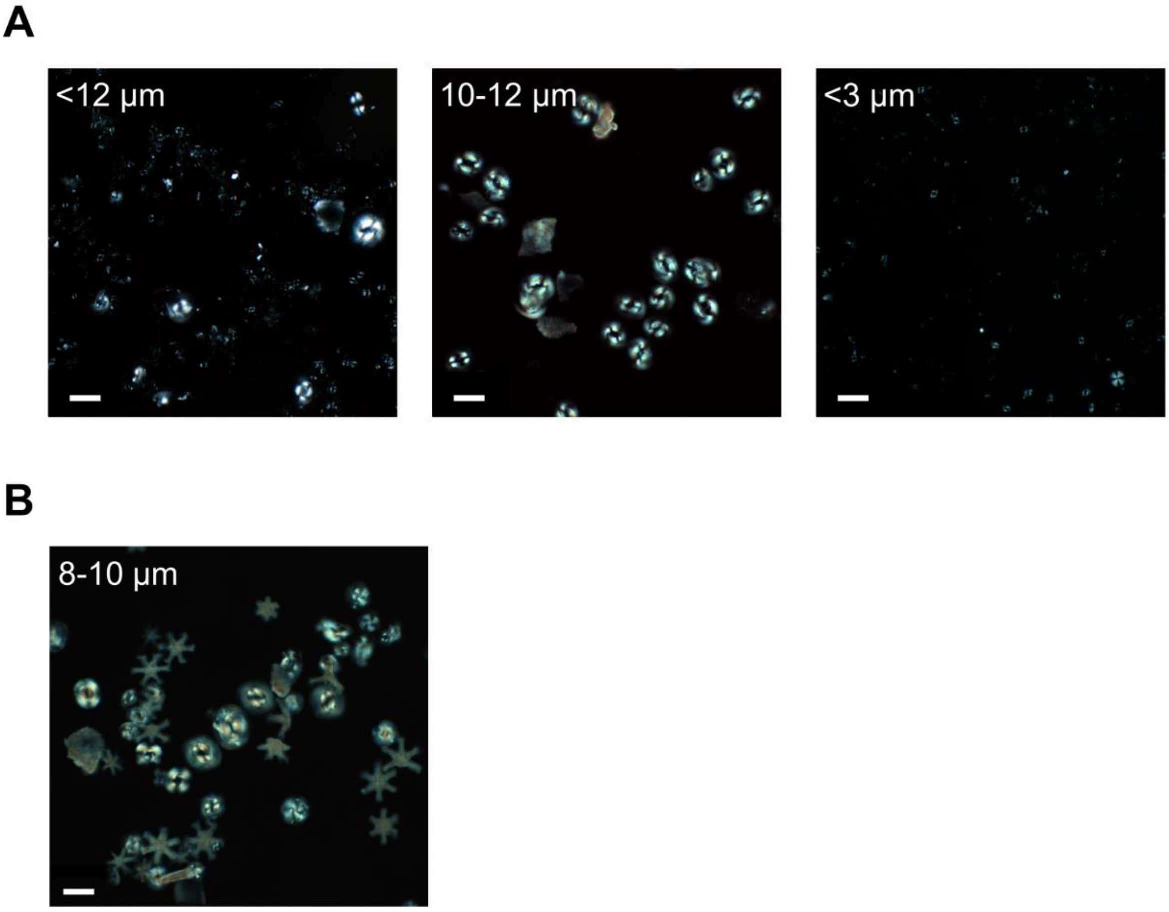

Supplementary Figure 1: Cross-polarized micrographs of size-separated fossil samples. (A) Cross-polarized micrographs of size-separated fractions from the Upper Pleistocene fossil sample (ODP Site 1123). Species composition for each fraction is summarized in Table S3. (B) Cross-polarized micrograph of the 8-10 µm size-separated fraction from the Langhian fossil sample (DSDP Site 588). The sample predominantly consists of Discoaster ssp. and *Coccolithus pelagicus*. Scale bars (bottom left) are 10 µm.

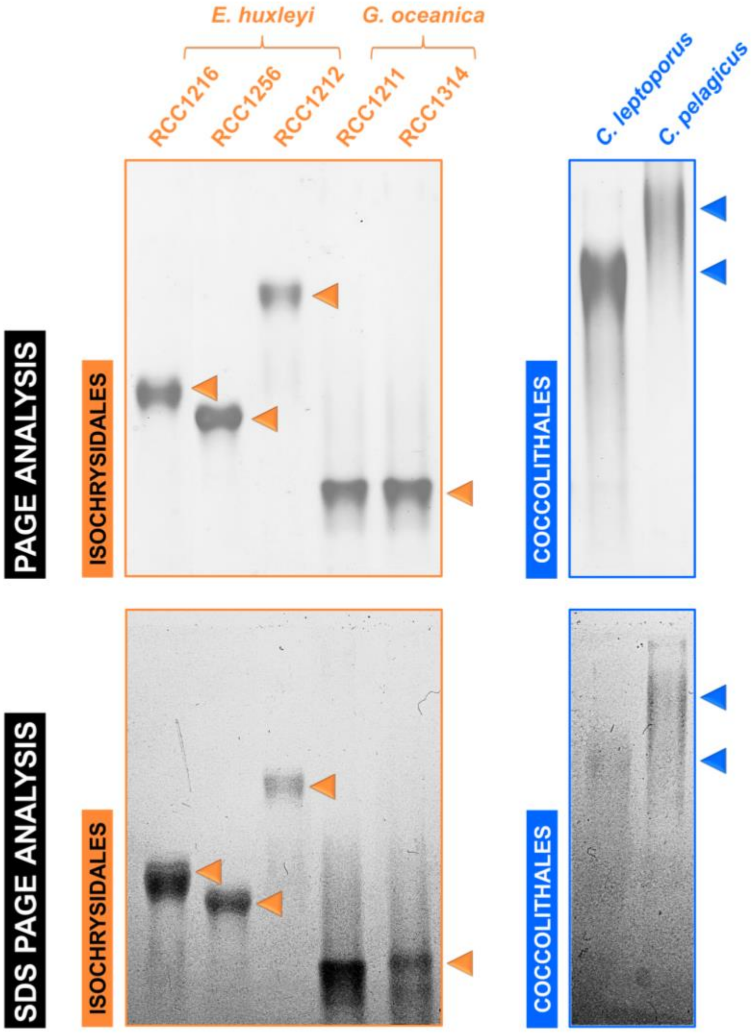

Supplementary Figure 2: Polyacrylamide gel electrophoresis (PAGE) analysis and denaturing Sodium Dodecyl Sulfate (SDS)-PAGE analysis of isolated coccolith-associated acidic polysaccharides (CAPs). PAGE (top) and SDS-PAGE (bottom) analysis were carried out using 4 µg of each CAP on 12% polyacrylamide gels. CAPs were stained with Alcian Blue. A single CAP is observed for all species/strains in both experiments; this is obvious from the presence of a single band in each lane indicated by an orange or blue arrowhead. The presence of the anionic detergent SDS during SDS-PAGE analysis impacts a negative charge on the CAPS which results in their migration only according to size. The fact that there is almost no discrepancy in the migration pattern of CAPs between PAGE and SDS-PAGE analysis means that the charged components of CAPs (like uronic and sulfonic acid residues) do not affect their electrophoretic mobility during PAGE analysis.

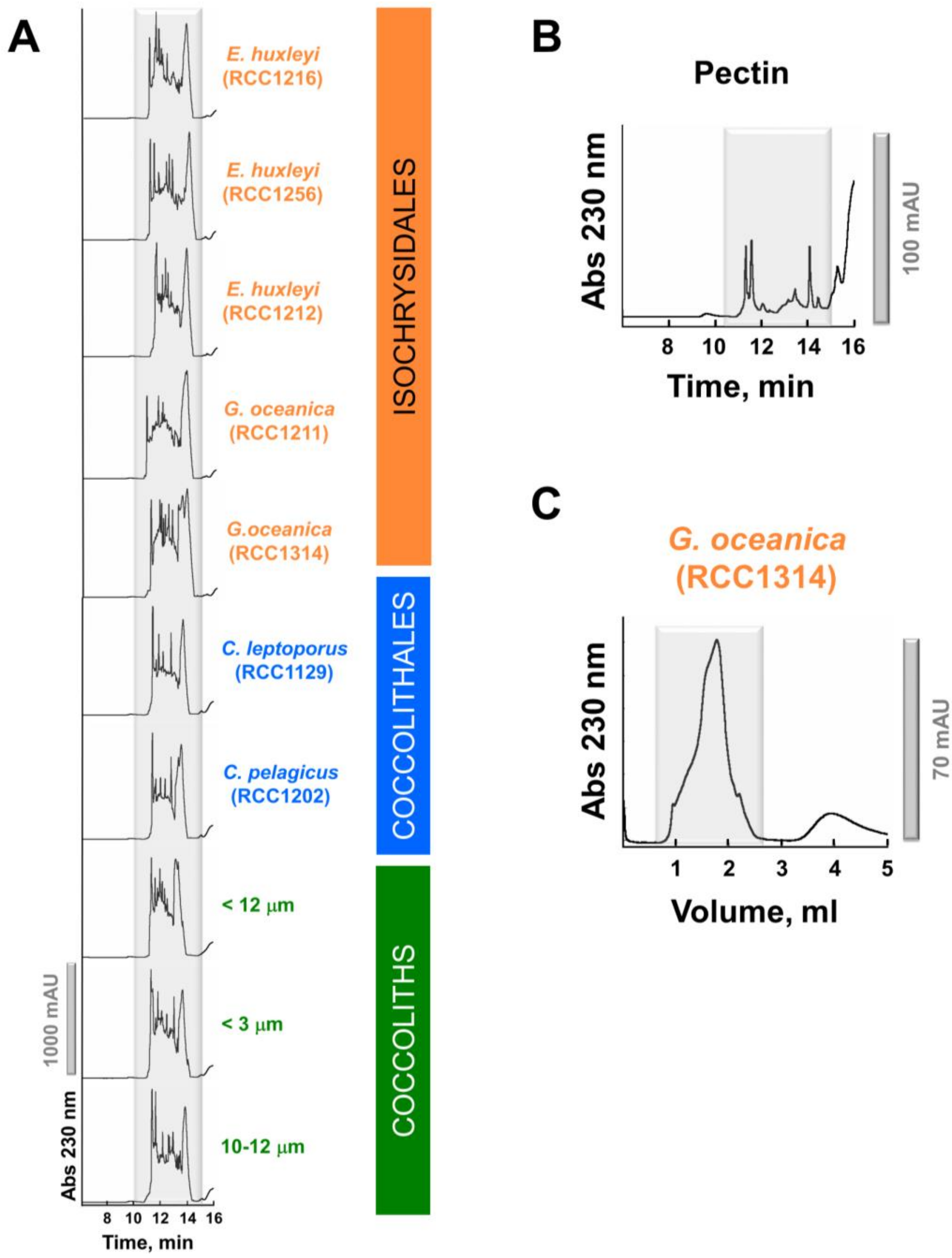

Supplementary Figure 3: Chromatographic analysis of isolated coccolith-associated acidic polysaccharides (CAPs). (A) Reverse-phase high performance liquid chromatography (RP-HPLC) profiles of isolated CAPs from cultured and fossil samples. ~100 µg of each CAP were loaded on a Vydac C8 column and their elution was detected at 230 nm. "mAU" refers to arbitrary absorbance units; their value correlates with the amount of CAP used for the analysis. Identical amounts of CAP have the same molecular polarity as they elute in the same solvent gradient range (marked by the grey shaded area). The occurrence of several peaks in this solvent gradient range indicates the presence of potential conformers for each CAP. (B) RP-HPLC profile of ~50 µg of pure pectin from apple (76282, Sigma-Aldrich), a known acidic polysaccharide. All isolated CAPs show similar polarity to apple pectin, because they elute in the same solvent gradient range (marked by the grey shaded area). (C) Representative size-exclusion chromatogram of the *G. oceanica* (RCC1414) CAP using a Superdex 200 PC 3.2/30 column. The single but broad peak in this chromatogram (marked by a grey shaded area) also indicates the presence of potential conformers for this CAP.

Supplementary Table 1: Cultured samples used in this study. Color-coding, where relevant, follows the color-coding used in the figures presented in this work. Species/strains were obtained from the Roscoff culture collection (www.sb-roscoff.fr/Phyto/RCC). The assigned species and morphotypes were confirmed by analysis of coccolith morphometry using the method of Young *et al.* (1) and guidelines from the Nannotax3 website (http://ina.tmsoc.org/Nannotax3/index.php?dir=Coccolithophores).

Species	Species code	Location	Morphotype
Emiliania huxleyi	RCC1216	Tasman Sea	R
Emiliania huxleyi	RCC1256	Icelandic Coast	A
Emiliania huxleyi	RCC1212	South Atlantic	B
*Gephyrocapsa oceanica**	RCC1211	Portuguese Coast	-
Gephyrocapsa oceanica	RCC1314	Bay of Biscay	-
Calcidiscus leptoporus	RCC1129	South Atlantic	-
Coccolithus pelagicus ssp. braarudii	RCC1202	Portuguese Coast	-

*RCC1211 was supplied from the culture collection as *Emiliania huxleyi* (morphotype B/C). We demonstrated that RCC1211 is *Gephyrocapsa oceanica* based on the migration pattern of the isolated coccolith-associated acidic polysaccharide (CAP) (Fig. 2A) and its uronic acid content (UAC) value (UAC of RCC1211: 2.19 µg / 4 µg of total CAP, UAC of RCC1314: 2.14 µg / 4 µg of total CAP). This result was also validated by traditional morphometric observation of the coccolith (Fig 1D).

Supplementary Table 2: Fossil samples used in this study. Color-coding, where relevant, follows the color-coding used in the figures presented in this work. Sedimentary material from the Upper Pleistocene was separated into mono-specific coccolith fractions (the quality of separation is summarized in Table S3 and cross-polarized micrographs of the size-separated fractions are shown in Fig. S1A). Samples from the Neogene period (DSDP Site 588) are almost mono-specific coccolith fractions with sizes larger than 8 µm, which predominantly consist of *Discoaster* ssp. and *Coccolithus pelagicus* (a representative cross-polarized micrograph of the Langhian fossil fraction is shown in Fig. S1B). Samples from the Late Cenomanian and Early Toarcian were not size separated and, according to literature, consist of *Watznaueria* ssp. and *Crepidolithus* ssp., respectively. Samples from the Campanian and Tithonian were not size separated and also mainly consist of larger coccolithophores (predecessors of the modern Coccolithales).

Geological timescale	Period	Age (Ma)	Sample origin	Reference
Upper Pleistocene	Quaternary	~ 0.107	ODP Site 1123, Chatham Rise, New Zealand	-
Serravalian	Neogene	~ 12.4	DSDP Site 588, Southwest Pacific	(2)
Serravalian	Neogene	~ 13.6	DSDP Site 588, Southwest Pacific	(2)
Langhian	Neogene	~ 14.5	DSDP Site 588, Southwest Pacific	(2)
Burdigalian	Neogene	~ 16.0	DSDP Site 588, Southwest Pacific	(2)
Campanian	Cretaceous	~ 76	SHUQ 325, Shuqualak-Evans bore hole, Mississippi, USA	(3)
Campanian	Cretaceous	~ 80	SHUQ 635, Shuqualak-Evans bore hole, Mississippi, USA	(3)
Late Cenomanian	Cretaceous	~ 94	Eastbourne, England	(4)
Tithonian	Jurassic	~ 150	BH1, Section 75/76, Whitestone Band, Kimmeridge Clay Formation, Dorset, England	(5,6)
Tithonian	Jurassic	~ 150	BH1, Section 76, Whitestone Band, Kimmeridge Clay Formation, Dorset, England	(5,6)
Early Toarcian	Jurassic	~ 180	Sancerre core, Paris Basin, France	(7)

Supplementary Table 3: Relative mass abundance and species composition of the separated fractions from the Upper Pleistocene fossil sample from the ODP Site 1123. Color-coding, where relevant, follows the color-coding used in the figures presented in this work.

Species	<12 µm	<3 µm	10-12 µm
Emiliania huxleyi	63.8%	80.2%	3.4%
Gephyrocapsa spp.	21.7%	17.3%	0.6%
Calcidiscus leptoporus	5.8%	2.5%	6.8%
Helicosphaera carteri	3.9%	-	2.3%
Coccolithus pelagicus	4.8%	-	87.1%

Supplementary Table 4: Yield of the extracted coccolith-associated acidic polysaccharides (CAPs). Total CAP was determined using the phenol-H_2SO_4 method of Hodge & Hofreiter (8). Color-coding, where relevant, follows the color-coding used in the figures presented in this work.

Sample	Geological timescale	Sample size	CAP (µg)
Emiliania huxleyi (RCC1216)	modern	1.2×10^9 cells	625.56
Emiliania huxleyi (RCC1256)	modern	9.0×10^9 cells	5500.23
Emiliania huxleyi (RCC1212)	modern	1.3×10^9 cells	297.35
Gephyrocapsa oceanica (RCC1211)	modern	1.1×10^9 cells	302.02
Gephyrocapsa oceanica (RCC1314)	modern	1.0×10^9 cells	375.21
Calcidiscus leptoporus (RCC1129)	modern	1.5×10^8 cells	94.77
Coccolithus pelagicus (RCC1202)	modern	6.9×10^7 cells	36.14
ODP Site 1123 (<12 µm)	Upper Pleistocene	9.216 g sediment	544.97
ODP Site 1123 (<3 µm)	Upper Pleistocene	8.535 g sediment	543.65
ODP Site 1123 (10-12 µm)	Upper Pleistocene	0.203 g sediment	19.84
DSDP Site 588 (12.4 Ma) (8-20 µm)	Serravalian	0.243 g sediment	30.21
DSDP Site 588 (13.6 Ma) (12-20 µm)	Serravalian	0.013 g sediment	5.51
DSDP Site 588 (14.5 Ma) (8-10 µm)	Langhian	0.344 g sediment	10.11
DSDP Site 588 (16.0 Ma) (10-12 µm)	Burdigalian	0.056 g sediment	54.75
Bulk, Shuqualak-Evans bore hole, Mississippi, USA (SHUQ 325)	Campanian	1.250 g sediment	4.00
Bulk, Shuqualak-Evans bore hole, Mississippi, USA (SHUQ 635)	Campanian	1.590 g sediment	12.00
Bulk, Eastbourne, England	Late Cenomanian	13.800 g sediment	9.61
Bulk, Kimmeridge Clay Formation, Dorset, England (BH1, Section 75/76)	Tithonian	18.100 g sediment	5.53
Bulk, Kimmeridge Clay Formation, Dorset, England (BH1, Section 76)	Tithonian	18.800 g sediment	9.40
Bulk, Sancerre Core, Paris Basin, France	Early Toarcian	7.300 g sediment	5.05

Supplementary Table 5: Overview of the characteristics of the coccolith-associated acidic polysaccharide (CAP), the coccolith and the coccolithophore. Color-coding, where relevant, follows the color-coding used in the figures presented in this work.

	COCCOLITHALES	ISOCHRYSIDALES		
	C. pelagicus	RCC1216	RCC1256	RCC1212
Morphotype	-	R	A	B
Number of CAP	One	One	One	One
Relative size of CAP	Large	Small	Small	Large
UAC/Total CAP	27%	60%	40%	22%
PIC rate (pg PIC/cell/hour)*	8.92-10.67	0.36-0.51	0.42-0.60	0.25-0.40
PIC/POC*	1.50-1.70	0.66-1.02	0.72-0.82	0.58-0.89
CAP production (pg/hour/cell)†	0.011	0.040	0.047	0.018
CAP:$CaCO_3$ (per coccolith)‡	0.016%	2.82%	4.85%	-
Coccosphere diameter (µm)**	20.0	4.73	4.93	5.52
Coccolithophore SA/V	0.30	1.27	1.21	1.09
Coccolith diameter (µm)§	10-16	3-4	3-4	3.5-5
Coccolith SA/V¶	5.89		5.31	

*PIC rate and PIC/POC values were obtained from Langer *et al.* (9) or measured according to (9). †CAP production rates were determined based on a rate of coccolith production of 1 coccolith/hour for Isochrysidales (10) and 1 coccolith/3 hours for *C. pelagicus* (11); the average number of coccoliths/cell in *E. huxleyi* and *C. pelagicus* is 13 and 15, respectively (12, 13). ‡$CaCO_3$ values were obtained from Cubillos *et al.* (14) or measured according to (14). **Coccosphere diameter was obtained from Hermoso *et al.* (15), Rickaby *et al.* (16) or measured using a Coulter Counter Z2 (Beckman-Coulter USA) fitted with a 50 µm aperture tube. §Coccolith diameter was obtained from Poulton *et al.* (12) and Cubillos *et al.* (14). ¶Coccolith SA/V was calculated with values from Young & Ziveri (17), Hassenkam *et al.* (18) and Bach *et al.* (19). (UAC stands for uronic acid content, PIC for particulate inorganic carbon, POC for particulate organic carbon. SA for surface availability and V for volume.)

Supplementary Table 6: Average uronic acid content (UAC) of the isolated coccolith-associated acidic polysaccharides (CAPs) from cultured Isochrysidales and Coccolithales as well as fossil Coccoliths from the Upper Pleistocene as presented in Fig. 2B. Details on the cultured and fossil samples can be found in Tables S1 and S2, respectively. UAC was measured in 4 µg of CAP using a modified carbazole-H_2SO_4 assay (20). Color-coding, where relevant, follows the color-coding used in the figures presented in this work.

Sample	UAC (µg)	Log_{10} UAC
Emiliania huxleyi (RCC1216)	2.34	0.369
Emiliania huxleyi (RCC1256)	1.58	0.198
Emiliania huxleyi (RCC1212)	0.89	-0.050
Gephyrocapsa oceanica (RCC1211)	2.19	0.340
Gephyrocapsa oceanica (RCC1314)	2.14	0.330
Calcidiscus leptoporus (RCC1129)	0.97	-0.013
Coccolithus pelagicus (RCC1202)	1.06	0.025
ODP Site 1123 (<12 µm)	2.46	0.391
ODP Site 1123 (<3 µm)	2.28	0.358
ODP Site 1123 (10-12 µm)	1.46	0.164

Supplementary Table 7: Average uronic acid content (UAC) of the isolated coccolith-associated acidic polysaccharides (CAPs) from fossil and modern coccolithales as presented in Fig. 3A. Details on the cultured and fossil samples can be found in Tables S1 and S2, respectively. The experimental UAC points in Fig. 3A have been labelled 1-13 (in purple) and correlate with rows 1-13 of this table. UAC was measured in 4 µg of CAP using a modified carbazole-H_2SO_4 assay (20). Color-coding, where relevant, follows the color-coding used in the figures presented in this work.

	Sample	Geological timescale	Age (Ma)	UAC (µg)	Log_{10} UAC
1	Sancerre Core, France	Early Toarcian	~ 180	2.02	0.305
2	BH1, Section 76, England	Tithonian	~ 150	2.68	0.428
3	BH1, Section 75/76, England	Tithonian	~ 150	2.77	0.442
4	Eastbourne, England	Late Cenomanian	~ 94	2.81	0.449
5	SHUQ 635, USA	Campanian	~ 80	2.08	0.318
6	SHUQ 325, USA	Campanian	~ 76	2.04	0.310
7	DSDP Site 588 (10-12 µm)	Burdigalian	~ 16.0	0.71	-0.149
8	DSDP Site 588 (8-10 µm)	Langhian	~ 14.5	1.15	0.061
9	DSDP Site 588 (12-20 µm)	Serravalian	~ 13.6	0.84	-0.076
10	DSDP Site 588 (8-20 µm)	Serravalian	~ 12.4	0.81	-0.092
11	ODP Site 1123 (10-12 µm)	Upper Pleistocene	~ 0.107	1.46	0.164
12	*C. pelagicus* (RCC1202)	modern/cultured	-	1.06	0.025
13	*C. leptoporus* (RCC1129)	modern/cultured	-	0.97	-0.013

Supplementary Table 8: Average uronic acid content (UAC) of the isolated coccolith-associated acidic polysaccharides (CAPs) and photosynthetic rates for *Emiliania huxleyi* strains as published in Rickaby *et al.* (16) and presented in Fig. 3B. The abbreviations used for each *E. huxleyi* strain in Fig. 3B are given in the first column of this table (shaded orange). UAC was measured in 4 µg of CAP using a modified carbazole-H_2SO_4 assay (20). The photosynthetic rate P_{max} represents the maximum rate of net photosynthetic oxygen evolution, while P_{2000} is the photosynthetic rate at near ambient environmental conditions of 2000 µmol kg^{-1} dissolved inorganic carbon (DIC).

	**Clonal ID**	**Morph.**	**Location**	**UAC (µg)**	P_{2000}	P_{max}
**NS1**	D366 26-1	A	North Sea	1.15	15.3	99.6
**NS2**	D366 36-2	A	North Sea	1.94	30.8	83.3
**BB**	D366 80-4	A	Bay of Biscay	1.49	16.6	62.4
**SO1**	SO 14-2	A	South West of Western Australia	1.31	30.5	45.0
**SO2**	SO 21-2	A	South East of South Africa	1.38	12.8	91.5
**GS1**	ARC 27-1	A	Greenland Sea	1.49	27.3	65.4
**NIS**	ARC 68-2	B/C	North Icelandic Sea	0.93	3.1	41.7
**IC**	RCC1256	A	Icelandic Coast	1.28	7.1	26.3

SUPPLEMENTARY REFERENCES

1. Young, J. *et al.* A guide to extant coccolithophore taxonomy. Special Issue *J. Nannoplankton Res.* **1**, 1-125 (2003).

2. Pagani, M., Arthur, M. A. & Freeman, K. H. Miocene evolution of atmospheric carbon dioxide. *Paleoceanography* **14**, 273-292 (1999).

3. Linnert, C. *et al.* Evidence for global cooling in the Late Cretaceous. *Nat. Commun.* **5**, 4194 (2014).

4. Tsikos, H. *et al.* Carbon-isotpe stratigraphy recorded by the Cenomanian-Turonian Oceanic Anoxic Event: correlation and implications based on three key localities. *J. Geol. Soc. London* **161**, 711-719 (2004).

5. Morgans-Bell, H. S. *et al.* Integrated stratigraphy of the Kimmeridge Clay Formation (Upper Jurassic) based on exposures and boreholes in south Dorset, UK. *Geol. Mag.* **138**, 511-539 (2001).

6. Pearson, S. J., Marshall, J. E. A. & Kemp, E. S. The White Stone Band of the Kimmeridge Clay Formation, an integrated high-resolution approach to understanding environmental change. *J. Geol. Soc.* **161**, 675-683 (2004).

7. Hermoso, M., Le Callonnec, L., Minoletti, F., Renard, M. & Hesselbo, S. P. Expression of the Early Toarcian negative carbon-isotope excursion in separated carbonate microfractions (Jurassic, Paris Basin). *Earth Planet Sc. Lett.* **277**, 194-203 (2009).

8. Hodge, J. E. & Hofreiter B. T. in *Methods in Carbohydrate Chemistry, Vol. 1.* (eds Whistler, R. L., Wolfrom, M. L., BeMiller, J. N. & Shafizadeh, F.) (Academic Press, New York, 1962).

9. Langer, G. *et al.* Species-specific responses of calcifying algae to changing seawater carbonate chemistry. *Geochem. Geophy. Geosy.* **7**, Q09006, doi:09010.01029/02005GC001227 (2006).

10. Paasche, E. A review of the coccolithophorid *Emiliania huxleyi* (Prymnesiophyceae), with particular reference to growth, coccolith formation, and calcification-photosynthesis interactions. *Phycol.* **40**, 503-529 (2002).

11. Taylor, A. R., Russell, M. A., Harper, G. M., Collins, T. F. T. & Brownlee, C. Dynamics of formation and secretion of heterococcoliths by *Coccolithus pelagicus* ssp. *braarudii. Eur. J. Phycol.* **42**, 125-136 (2007).

12. Poulton, A. J., Young, J. R., Bates, N. R. & Bach, W. M. Biometry of detached *Emiliania huxleyi* coccoliths along the Patagonian Shelf. *Mar. Ecol. Prog. Ser.* **443**, 1-17 (2011).

13. Gibbs, S. J. *et al.* Species-specific growth response of coccolithophores to Palaeocene-Eocene environmental change. *Nat. Geosci.* **6**, 218-222 (2013).

14. Cubillos, J. C., Henderiks, J., Beaufort, L., Howard, W. R. & Hallegraeff, G. M. Reconstructing calcification in ancient coccolithophores: Individual coccolith weight and morphology of *Coccolithus pelagicus* (sensu lato). *Mar. Micropaleontol.* **92-93**, 29-39 (2012).

15. Hermoso, M., Horner, T. J., Minoletti, F. & Rickaby, R. E. M. Constraints on the vital effect in coccolithophore and dinoflagellate calcite by oxygen isotopic modification of seawater. *Geochim. Cosmochim. Ac.* **141**, 612-627 (2014).

16. Rickaby, R. E. M. *et al.* Environmental carbonate chemistry selects for phenotype of recently isolated strains of *Emiliania huxleyi*. *Deep-Sea Research Part II* **127**, 28-40 (2016).

17. Young, J. R. & Ziveri, P. Calculation of coccolith volume and its use in calibration of carbonate flux estimates *Deep-Sea Res. Pt. II* **47**, 1679-1700 (2000).

18. Hassenkam, T., Johnsson, A., Bechgaard, K. & Stipp, S. L. S. Tracking single coccolith dissolution with picogram resolution and implications for CO_2 sequestration and ocean acidification. *Proc. Natl. Acad. Sci.* **108**, 8571-8576 (2011).

19. Bach, L. T., Bauke, C., Meier, K. J. S., Riebesell, U. & Schulz, K. G. Influence of changing carbonate chemistry on morphology and weight of coccoliths formed by *Emiliania huxleyi. Biogeosciences* **9**, 3449-3463 (2012).

20. Cesaretti, M., Luppi, E., Maccari, F. & Volpi, N. A 96-well assay for uronic acid carbazole reaction. *Carbohyd. Polym.* **54**, 59-61 (2003).
